# Automatic Localization of the Left Ventricle from Cardiac Cine Magnetic Resonance Imaging: A New Spectrum-Based Computer-Aided Tool

**DOI:** 10.1371/journal.pone.0092382

**Published:** 2014-04-10

**Authors:** Liang Zhong, Jun-Mei Zhang, Xiaodan Zhao, Ru San Tan, Min Wan

**Affiliations:** 1 Bioengineering Department, National Heart Centre Singapore, Singapore, Singapore; 2 Cardiovascular & Metabolic Disorders Program, Duke-NUS Graduate Medical School Singapore, Singapore, Singapore; 3 Geometrical Modelling, Institute of High Performance Computing, A*STAR, Singapore, Singapore; University Medical Center (UMC) Utrecht, Netherlands

## Abstract

Traditionally, cardiac image analysis is done manually. Automatic image processing can help with the repetitive tasks, and also deal with huge amounts of data, a task which would be humanly tedious. This study aims to develop a spectrum-based computer-aided tool to locate the left ventricle using images obtained via cardiac magnetic resonance imaging. Discrete Fourier Transform was conducted pixelwise on the image sequence. Harmonic images of all frequencies were analyzed visually and quantitatively to determine different patterns of the left and right ventricles on spectrum. The first and fifth harmonic images were selected to perform an anisotropic weighted circle Hough detection. This tool was then tested in ten volunteers. Our tool was able to locate the left ventricle in all cases and had a significantly higher cropping ratio of 0.165 than did earlier studies. In conclusion, a new spectrum-based computer aided tool has been proposed and developed for automatic left ventricle localization. The development of this technique, which will enable the automatic location and further segmentation of the left ventricle, will have a significant impact in research and in diagnostic settings. We envisage that this automated method could be used by radiographers and cardiologists to diagnose and assess ventricular function in patients with diverse heart diseases.

## Introduction

Cardiovascular Disease (CVD) is currently the leading cause of death, killing 17.3 million people worldwide each year. This figure represents one-third of total death, a proportion that is still increasing. It is estimated that by 2030, CVD will kill 23.6 million people annually [1]. For clinicians, the examination and analysis of medical cardiac images is of great significance for diagnosis and treatment. The reliability of quantitative assessment of cardiac functions such as muscle deformation and ventricular ejection fraction is dependent on the precision and correctness of the heart chamber segmentation [2].

Usually, this segmentation task is performed manually via software assistance, which is quite time-consuming. One trained clinician can delineate one image in half a minute, and it would take him/her more than two hours to finish the whole data set of 11 slices by 25 frames. Furthermore, the results often present a high intra/inter clinician variability [3]. Over the past decade, automation of this tedious yet significant procedure has received a lot of attention from not only medical imaging but also the computer vision community [4]–[9]. Of the four heart chambers, the imaging of the left ventricle (LV) is of the greatest importance and interest due to its physiological fatality. Of the 70 studies surveyed in Petitjean and Dacher [3], 49 of them address the LV segmentation problem exclusively, with 18 solving segmentation of both ventricles.

One important stage in the automatic segmentation approach is to locate the left ventricle in the whole image and limit the computation domain within a vicinity of the left ventricle. The localization procedure is sometimes referred to as region of interest (ROI) identification. This procedure not only decreases the computational load, but also provides an initial guess for deformable segmentation approaches. Previously, it was preferred that the ROI identification be done manually by selecting the center of the ROI [10]–[12] or drawing a bounding contour. To make the whole segmentation procedure automatic, it will be necessary to automate LV localization as well.

The existing approaches for the automatic localization of the LV can be generally categorized into two groups. One group is pattern recognition approaches [13], which require a learning stage. The desired “donut” pattern is decomposed into a multi-dimensional feature vector, which is further modelled as a Markov process. The system is trained using both positive and negative examples identified manually. The trained system is utilized to classify the image pixels as the LV boundary [14]–[17]. The whole procedure is a specific application of pattern recognition, the success of which is dependent on a proper training example series as well as the relevancy between the training examples and the studied cases.

The other group is temporal dimensional approaches. These methods are based on the fact that the heart is the only organ with substantial motion in the field of view (FOV). In an ideal cine sequence, the image is stationery aside from the heart. The variance image is calculated from a 2 D+t original image sequence. The high intensity in the variance image corresponds to the vigorous movement in the original image, which usually happens at the endocardium and epicardium of both ventricles as well as at the septum. A circle Hough transform [18] is used to detect the potential circle approximating the endo/epi-cardium of the LV. Interested readers are referred to previous resources [19]–[23]. One alternative approach based on Fourier analysis has previously been proposed [24], [25].Instead of using the variance image to recognize the motion, the first Harmonic image is used. The circle Hough detection procedure is identical to that of the variance image approaches.

Compared to the pattern recognition methods, the temporal approaches do not require a large training set, which is also a tedious task. They also exploit the information in between the frames. However, both the variance image approach and Fourier analysis approach lack robustness and precision in locating the LV. The LV is not the only moving chamber in the FOV. The variance or the first harmonic image contains much more than the LV boundary and this could hardly be extinguished. The movement of the right ventricle (RV) would unavoidably lead to imprecise or even incorrect circle detection and ROI identification. A more detailed analysis and some examples are provided in Section 2.

In this study, short axis cardiac images at the basal location were prepared for ten normal volunteers. Harmonic images of all orders were examined visually and quantitatively. Two observations were seen regarding the LV and RV in Harmonic images as follows, based on which had more information inferred from Harmonic images of high order that could be utilized in a sophisticated way:

- In all Harmonic images except the zeroth one, the RV region presents higher brightness than the LV region.
- As the Harmonic order increases, the superiority of the RV to the LV increases.

Two Harmonic images were utilized, the first Harmonic image (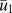) and a higher-order Harmonic image (the fifth Harmonic image (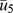) in our study). A new circle detector was designed based on the anisotropic weighted circle Hough transform. The 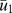 image with the most substantial LV visibility was an input of the detector with a positive weight, while 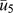 was the other input with a negative weight to suppress the RV interruption. This dual input Hough detector could largely eliminate the RV interruption, leading to a robust and precise LV localization.

The remainder of this article is organized as follows. Section 2 gives a brief review on earlier methods and shows their relation to Harmonic images. In Section 3, Harmonic images of all orders are analysed and two facts concerning the LV and RV are presented. An anisotropic weighted circle Hough detector is then proposed for LV localization. Section 4 shows the experimental results of the proposed localization method. Section 5 concludes the article.

## Related Work on Harmonic Images of Cine Magnetic Resonance Imaging

Several LV localization methods based on the variance image have been developed [19]–[23]. A similar approach based on Fourier analysis has been described [24], [25]. In this section, we first define the Harmonic images of a cine image sequence. We then provide a brief review of those earlier studies and a reformulation of them into the Harmonic image framework. At the end of the section, we present their limitations and illustrate these using a concrete example.

### Discrete Fourier Transform and Harmonic Images

Given a 2 D+t cine Magnetic Resonance (MR) image 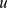 with the resolution of 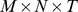, each pixel position over time presents a discrete time series, (i.e. 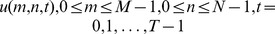). For the rest of the discussion in this section, we omit the spatial coordinates 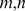 for simplicity, (e.g., 
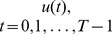
).

Discrete Fourier transform (DFT) of 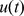 is as follows.
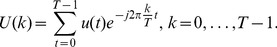
(1)


Inverse Discrete Fourier transform (IDFT) of 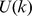 is as follows.
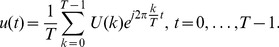
(2)



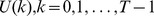
 presents the decomposition of the time sequence 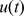 in term of frequency. We define the 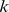-th harmonic series of 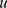 as IDFT to the 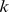-th frequency component separately as follows,
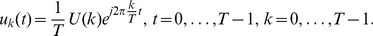
(3)


The 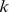-th Harmonic image is defined as the 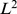-norm of the 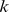-th harmonic series.
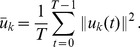
(4)


### Earlier Methods on Harmonic Images

The LV localization for cine images could be dated to early this century. Lin et al. and Jolly [24], [25] used the first Harmonic image 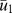 to detect LV. Others [19]–[23] provided similar detection methods based on the variance image. We will first recall the definition of the variance image and then show how it is related to the Harmonic images. Let 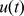 be the same as in Section 2.1. The mean image and the variance image of 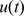 are defined as follows.
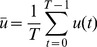
(5)

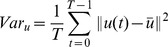
(6)


The equivalence between the mean and the direct current (dc) image, (i.e., the 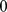-th harmonic image) is also obvious:
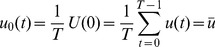
(7)


The relation between the variance image and other harmonic images could be derived as well.
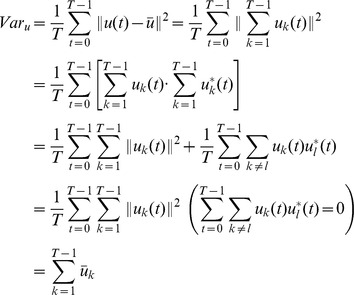



As the above derivation shows, the variance image is the sum of Harmonic images of all orders except the zeroth order (i.e., the dc image or the mean image).

**Figure 1:**
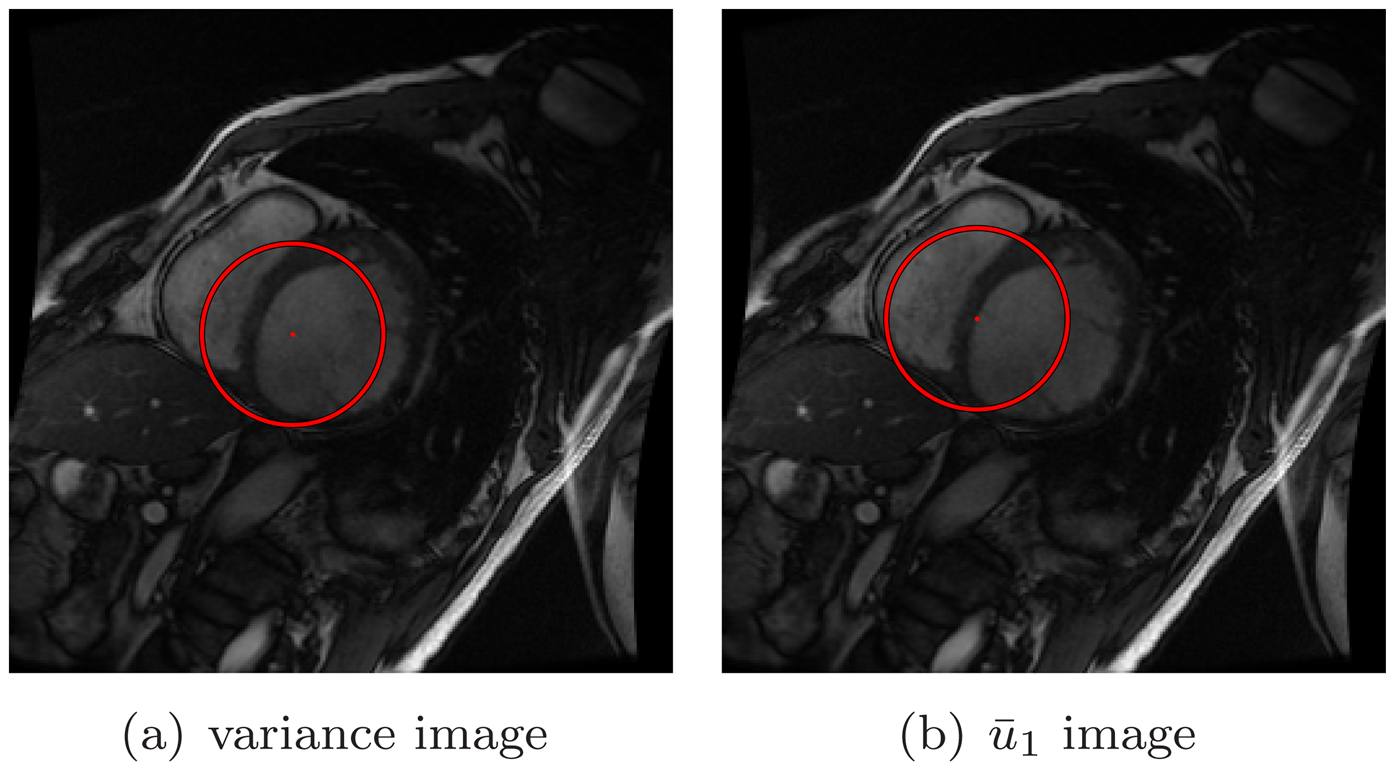
The imperfect detection.

Most of the earlier methods based on Harmonic images have one disadvantage, the RV interruption. The Harmonic images reflect the organ motion pattern from the spectrum aspect. Motions of both ventricles are present in he Harmonic images. Hence, the precision of LV detection would be undermined by RV interruption. Such a limitation could be observed in most of the earlier studies that we mentioned [19]–[25]. Figure 1 shows an imperfect detection using 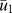 and 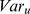.

**Figure 2:**
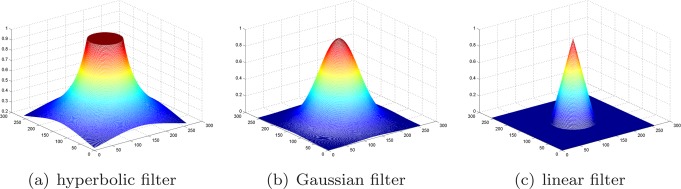
The variance image and the first harmonic image.

To tackle the RV interruption, some earlier studies made the assumption that the LV is located around the center of the FOV [26], [27]. Based on this assumption, a bell shaped or cone shaped filter centered in the FOV is applied to the detection procedure. Figure 2 shows several options regarding the filter shape. However, the aforementioned assumption is strongly relevant to the scanning operator or the participant. The variability of both would cause the assumption to no longer hold. This limitation motivates our study.

## Methods

In this study, we tested algorithm on 10 healthy volunteers. This study was approved by the Singhealth Centralised Institutional Review Board (CIRB No: 2009/705/C) for human research. All enrolled participants gave written informed consent. The MR data are deposited in hospital and are available for research and education purpose. Cardiac relating measurements for each volunteer were give in Table 1.

**Table 1:** Statistics on Subjects.

Volunteer	height	weight	BSA	LV mass	RR	SBP	DBP	LVEDV
	(m)	(kg)	( 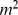 )	(g)	(ms)	(mmHg)	(mmHg)	(ml)
1	1.71	68.2	1.8	102	745	115	63	130
2	1.8	97.5	2.2	125	1005	123	72	202
3	1.66	78	1.9	115	780	136	79	125
4	1.56	59.2	1.6	78	765	111	60	100
5	1.53	46.5	1.4	66	830	110	67	101
6	1.47	47.1	1.4	85	880	117	75	125
7	1.62	66	1.7	120	1085	126	78	151
8	1.47	55	1.5	67	860	92	55	98
9	1.6	49.8	1.5	82	1000	100	63	136
10	1.74	77.7	1.9	105	985	128	77	149
Average	1.616	64.5	1.69	94.5	893.5	115.8	68.9	131.7
**Volunteer**	**LVESV**	**LVSV**	**LVEF**	**LVMassindex**	**RVEDV**	**RVESV**	**RVSV**	**RVEF**
	**(ml)**	**(ml)**	**(** 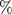 **)**	**(** 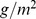 **)**	**(ml)**	**(ml)**	**(ml)**	**(** 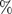 **)**
1	57	73	56	57	169	86	83	49
2	79	123	61	57	237	110	127	54
3	52	73	59	61	161	87	73	46
4	30	70	70	49	105	40	65	62
5	32	69	68	47	105	40	65	62
6	36	89	71	61	155	69	86	55
7	47	104	69	70	177	78	99	56
8	32	66	67	45	127	58	69	54
9	70	66	48	57	117	61	56	48
10	58	91	61	54	160	72	88	55
Average	49.3	82.4	63	55.75	151.3	70.1	81.1	54.1

### Two Observations of the LV and RV in Harmonic Images

**Figure 3:**
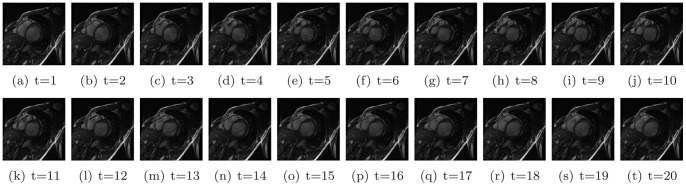
All frames of a cine image.

**Figure 4:**
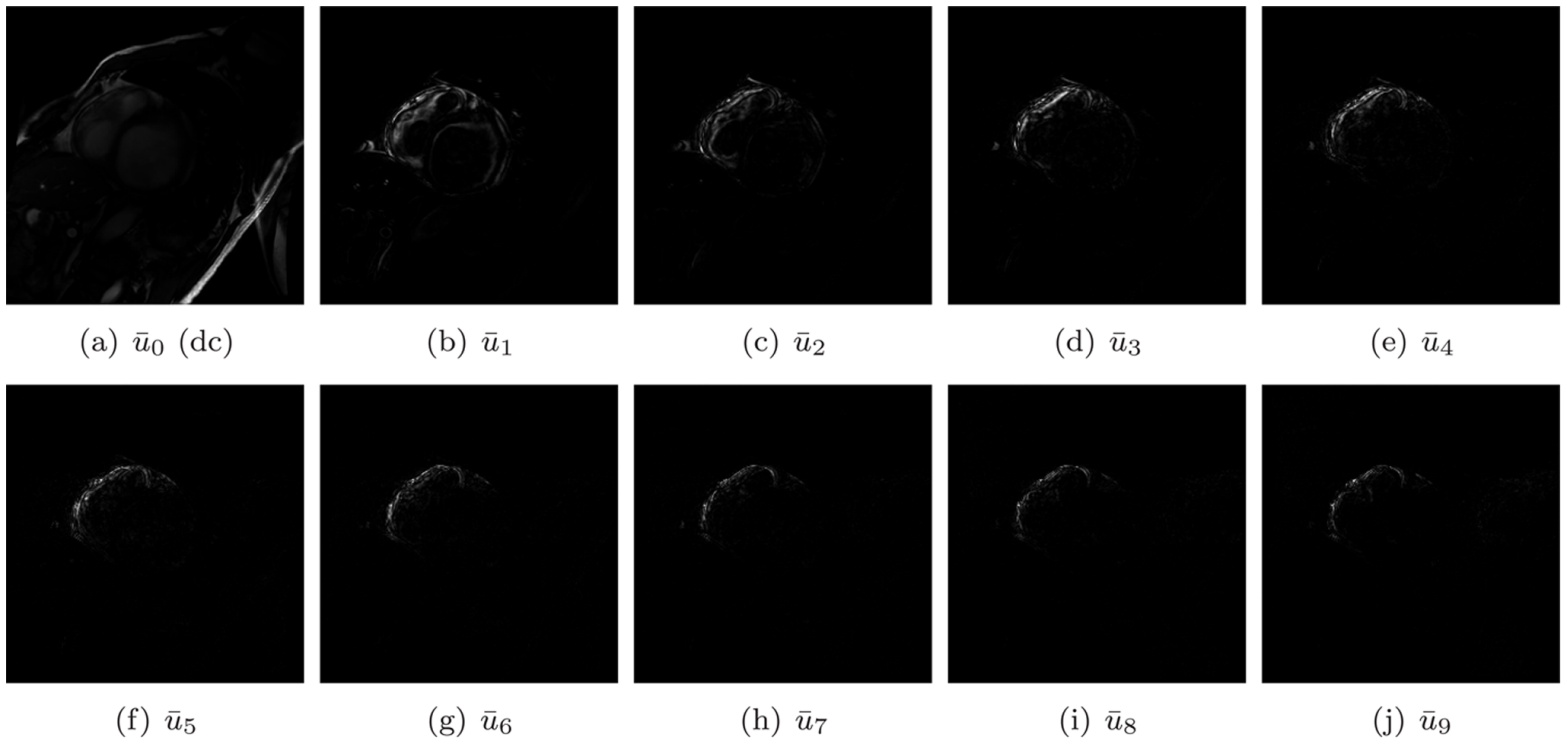
All Harmonic images of the cine image.

**Figure 5:**
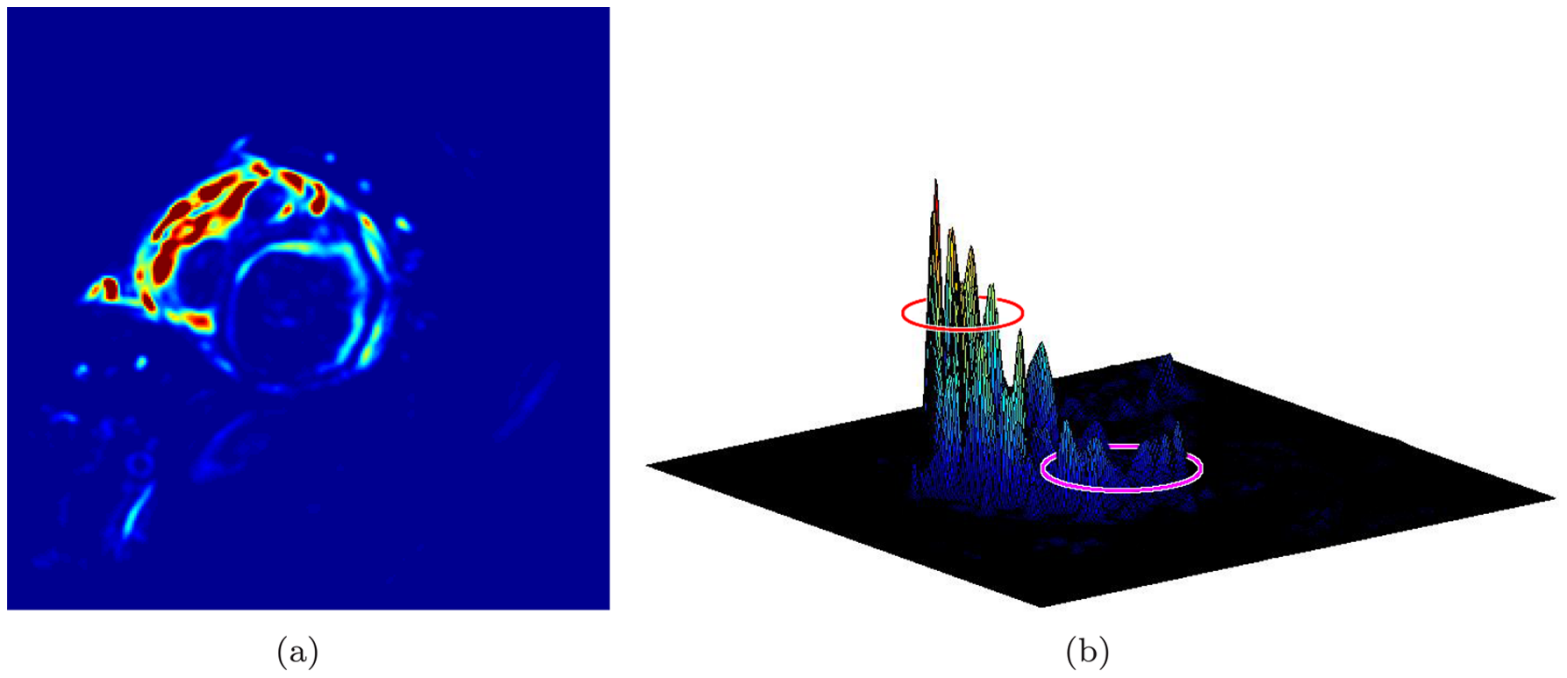
The variance image and the first harmonic image.

In this study, we developed a new LV localization method based on Harmonic images and an anisotropic circle Hough transform. First we examined the different patterns of the LV and RV in Harmonic images. An example containing 20 frame cine MR images is illustrated in Figure 3. The Harmonic images from the zeroth to the ninth order were generated as shown in Figure 4. We gave the first Harmonic image a close examination. The color-rendered visualization of 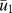 and the surface plot are shown in Figure 5(a) and (b), respectively. The LV and RV regions are circled. We could observe that the left “peak” (RV region) was higher than the right “peak” (LV region). The RV region had a larger number of brighter pixels in 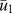.

**Figure 6:**
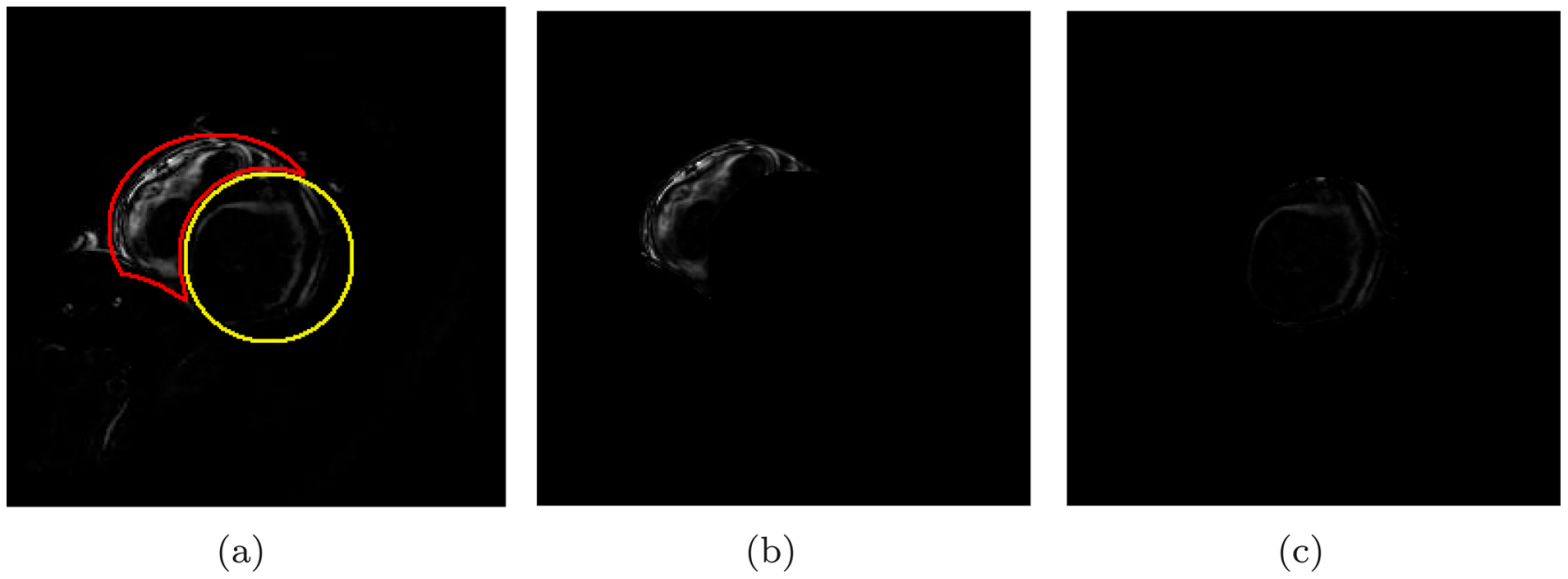
Predefined RV and LV region as well as masked images.

**Figure 7:**
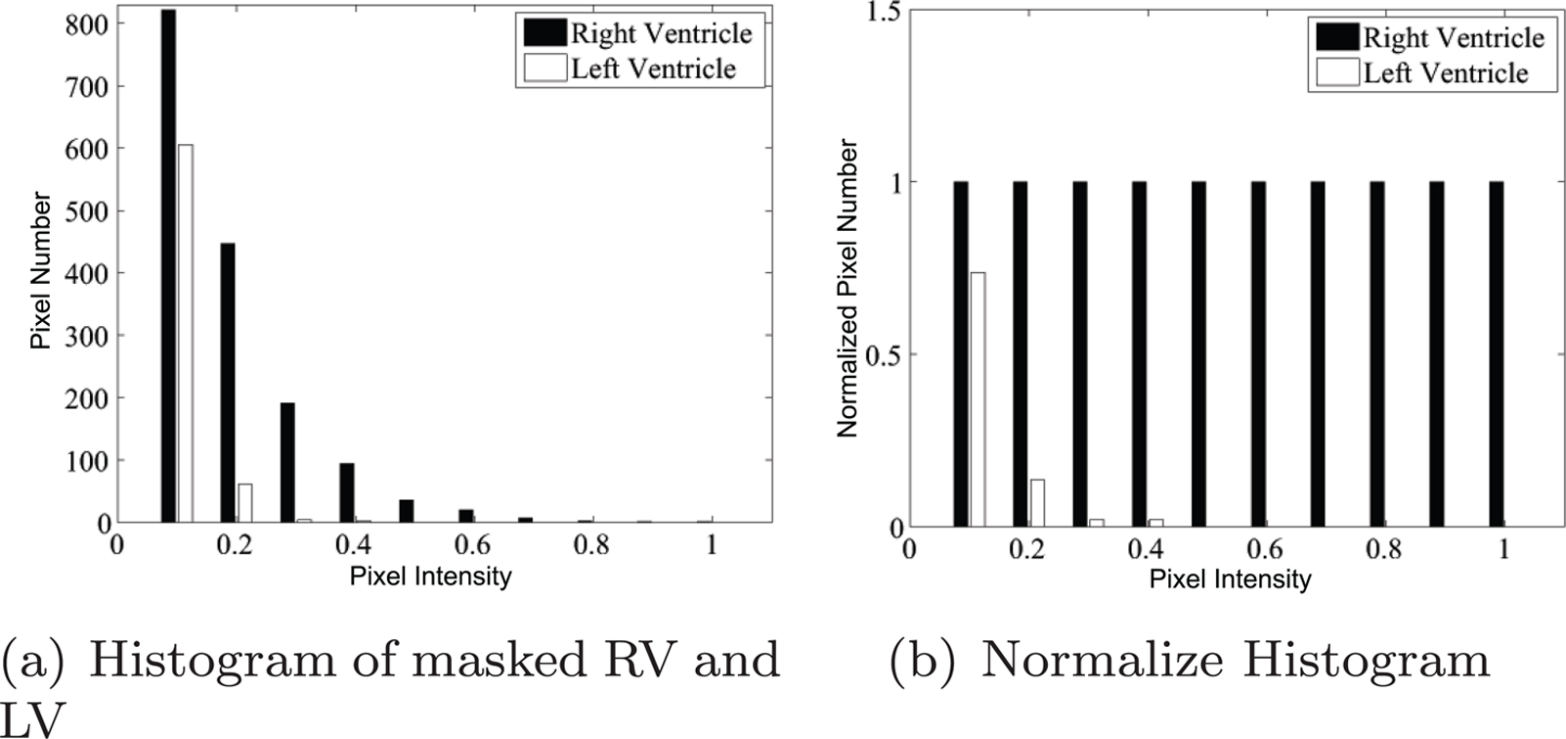
Histogram of RV and LV in 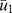 image.

We quantified the observed result beyond the observation and visual analysis. The LV and RV regions were identified manually in 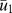, see Figure 6(a). The masked RV and LV images are shown in Figure 6(b) and (c), respectively. The statistics of these two regions were computed. The histogram of each is shown in Figure 7 along with a normalized version. From the histograms, it could be observed that the LV region had fewer pixels with high brightness. We present the first observation:

#### Observation 1

In the first Harmonic image, the RV region presents higher brightness than the LV region.

This observation explains the limitation of earlier studies. If the circle Hough detection is carried out upon the first Harmonic image or the variance image, the brighter RV region would interrupt the LV detection. This observation also implies that the detected circle tends to shift toward the RV region (Figure 1).

**Figure 8:**
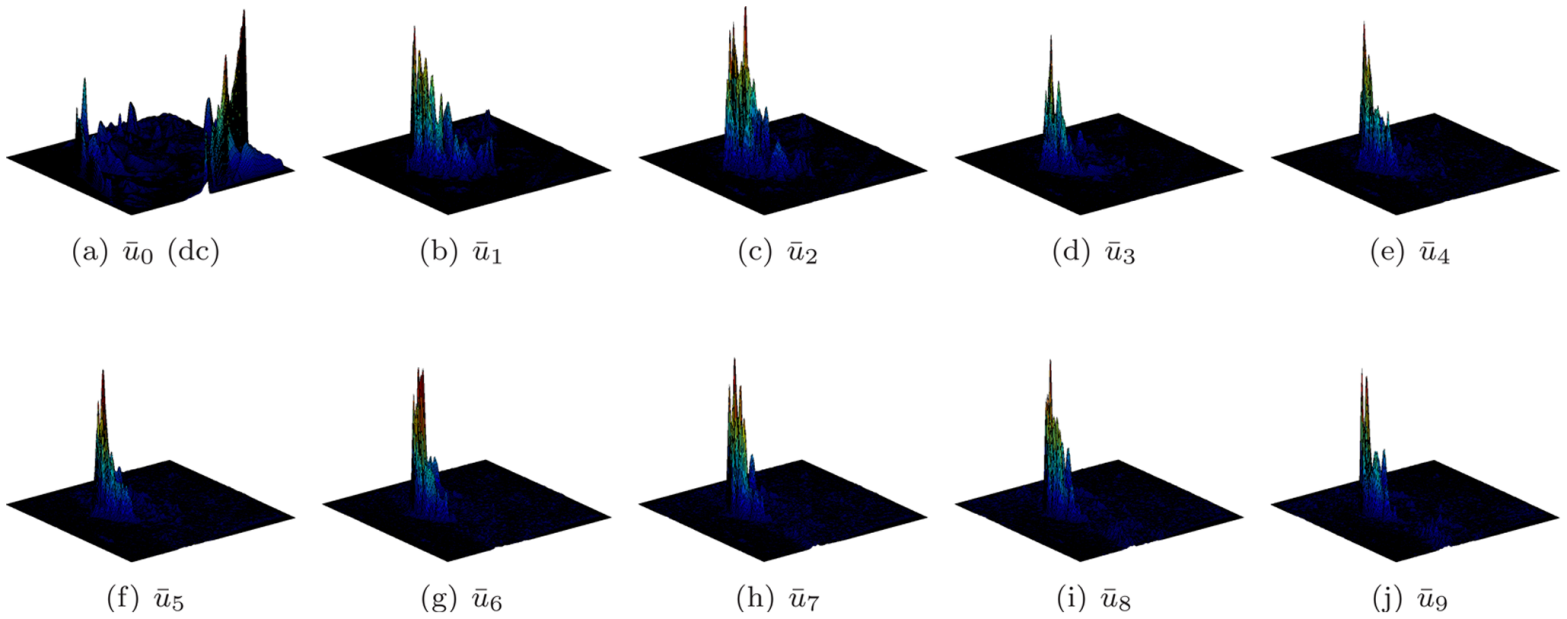
Surface plots of all harmonic images.

**Figure 9:**
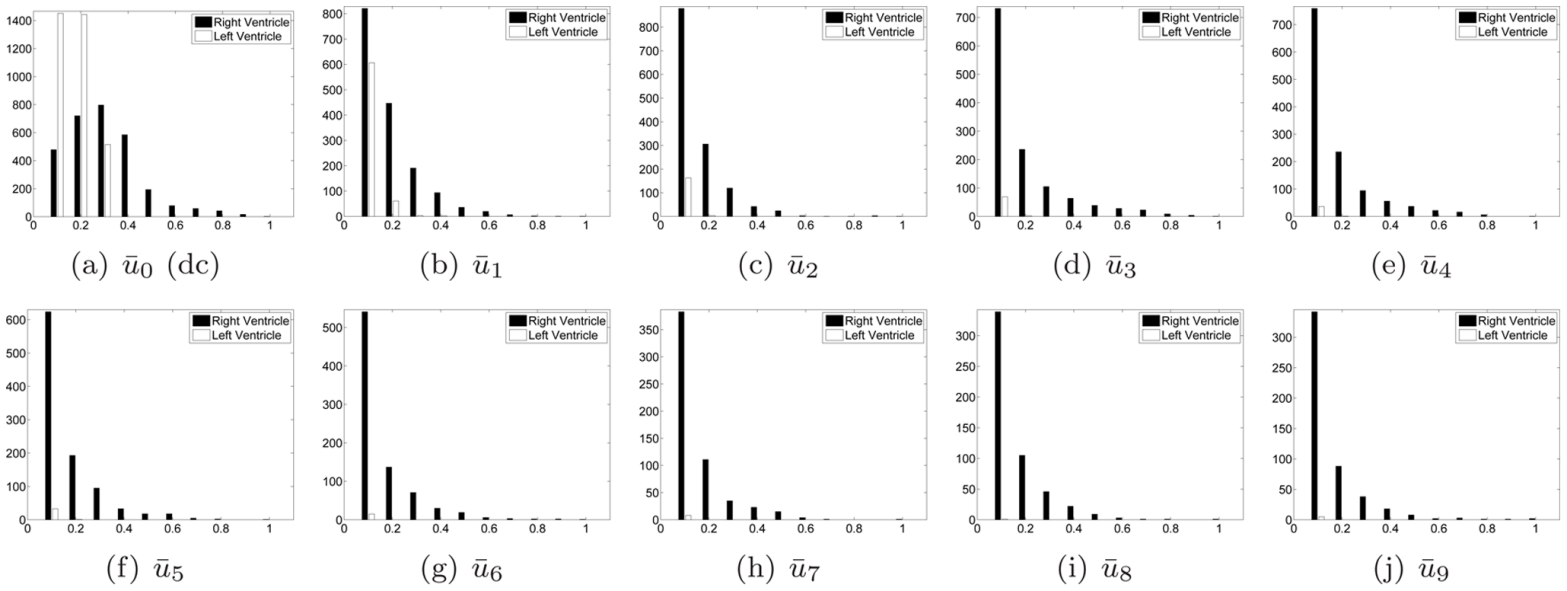
Histograms of all harmonic images.

The observation represents the superiority of the RV to the LV in the first Harmonic image. We next inspected the existence of such superiority in other Harmonic images. Surface plots are shown in Figure 8 and the histograms are shown in Figure 9. The superiority of the RV was evaluated in all Harmonic images except the zeroth Harmonic image, i.e., 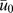. Observation 1 could then be extended as follows:

#### Observation 1

In all Harmonic image except 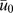, the RV region presents higher brightness than the LV region.

It could also be observed from Figure 8 that the LV peaks diminish quickly as the Harmonic order increases. From the fourth harmonic image, the LV could hardly be observed from either the image or the surface plot. Meanwhile, RV peaks could be observed in the first through the last harmonic images. This trend could also be verified by the diminishing white bars in Figure 9.

**Figure 10:**
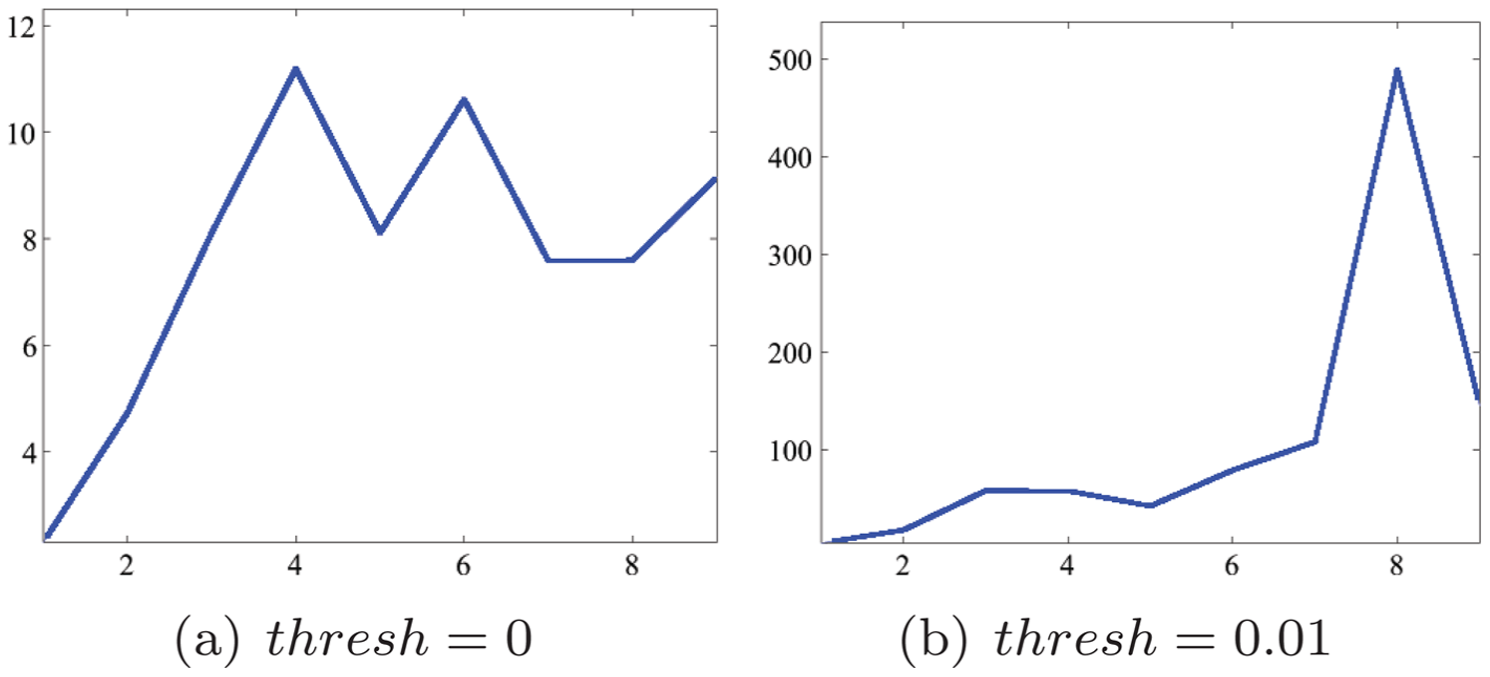
ARLR of different 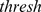 parameter.

To quantize this trend, we defined an index of Average RV to LV ratio (ARLR) as follows. With the RV and LV regions, 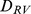 and 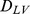, as well as a threshold parameter 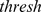, the average of all observable pixels (brighter than 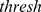) within 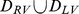 is calculated. Then the pixels that had a higher intensity than this average within the two regions were counted, respectively. The ratio of the two count numbers was called ARLR. Mathematically, this index is defined as follows:
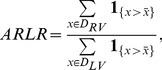
(8)where



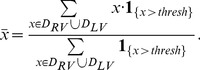
(9)The plot of ratio against harmonic order is shown in Figure 10. A rising trend could be found from the curve for different threshold parameters. From this comes our second observation:

#### Observation 2

As the Harmonic order increases, the superiority of the RV to the LV increases.

From the visualization and quantitative analysis in the previous subsection, we could draw the conclusion that the RV motion involves more high frequency motion than does the LV motion. Regarding the observation for this special cardiac motion pattern, we could provide the following explanation. Covered by the supporting pericardium, the heart lies close to the middle of the breast-bone. The heart position is oblique. The inferior wall of the LV lies against the diaphragm, while the lateral wall and the anterior wall of the LV lie against the left lung. In contrast, the RV has less support. The superior and anterior position of the RV relative to the LV results in less support from the diaphragm and no support from the lungs. When pumping, the LV moves with the everlasting resistance of the stationary lungs and diaphragm (when the participant holds his/her breath). This causes the self-oscillation frequency of the LV to decrease, thus lowering the high frequency portion. In contrast, the RV motion contains more high frequency motion due to it being relatively free of burden.

### Anisotropic Weighted Circle Hough Transform

In this subsection, we first introduce the classic circle Hough transform. We next present two modifications on the algorithm, as well as their advantages.

#### Classic circle hough transform

Given an edge map derived from an image containing potential circles, the classic circle Hough transform is described as follows. A parameter space is constructed at the same size as the original image. Each pixel in the parameter space has an accumulator, all of which are initialized to zero. Provided an estimate of the radius, 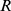, and a pixel 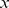 in the edge map, with an intensity larger than the threshold, one is added to all accumulators that intersect with the circle centered at 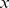 with a radius of 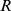. After completing this voting procedure with respect to every point on the edge map, the highest accumulator is considered to be the center of the detected circle with radius 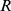.

When a good estimate of the radius 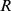 is not available, an interval of possible radius is provided instead. There are usually two options to adapt the circle Hough transform. 1) Repeat the above voting procedure with respect to every 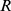 in the interval. After all loops of voting, the final winning accumulators suggest the circle center with the radius of the mean of the interval. 2) Instead of a 2 D parameter space, the point 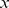 votes into a 3 D parameter space. The extra dimension is for the radius parameter and the size is the length of the radius interval. The final winning accumulator provides not only the center but also the radius of the detected circle.

#### Anisotropic weighted circle hough transform

**Figure 11:**
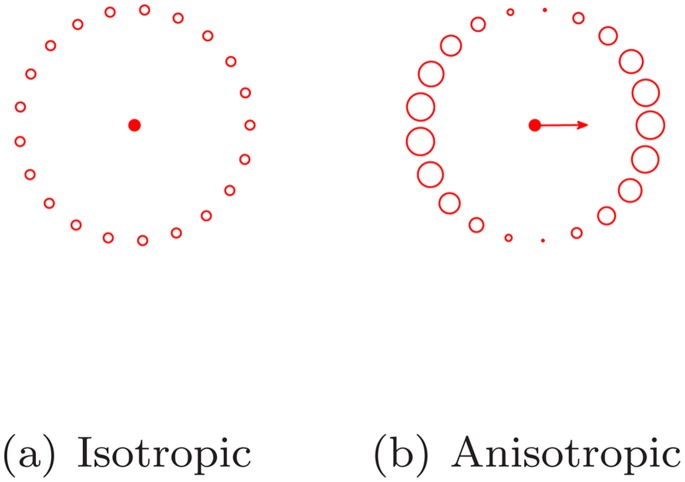
Two circle Hough transform.

In the classic circle Hough transform, the point 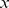 votes to all candidates on the circle 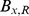, equally. This kind of circle Hough transform is isotropic. See Figure 11(a), in which the diameters of the candidate rings stand for the voting weights.

However, the probabilities of the target center falling into the candidates on the circle are not the same. If the gradient map of the original image is available as well, the gradient at 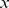 could provide more precise voting if one imagines the gradient vector points to the target center. The voting into the candidates is weighted according to the extent the candidate deviates from the direction the gradient vector points. In our study, we use cosine of the deviation angle to determine the weight. If the candidate is parallel to the gradient vector, the voting weight is maximized; if it is orthogonal to the gradient, the voting weight is zero. See Figure 11(b).

**Figure 12:**
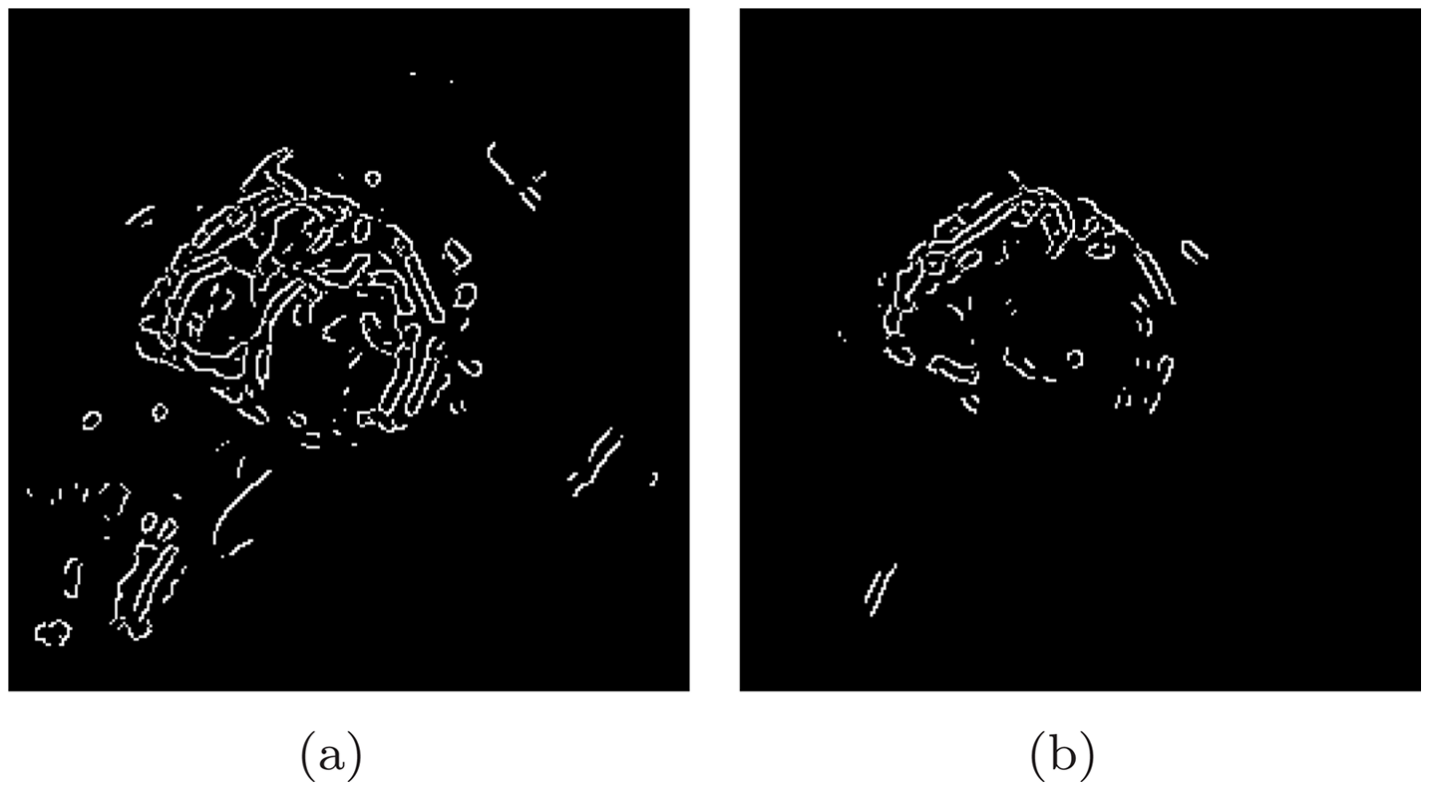
Edge maps 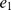 and 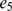 from 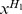 and 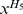.

Considering the discussion on the increasing ARLR with the harmonic order and our goal of identifying the LV, we use two harmonic images, 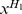 and 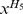 to cooperate in the circle detection. Both of these images are passed to the edge detector. Two edge maps 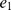 and 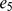 are obtained, as are the gradient maps 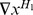 and 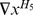 as the by-product. The 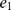 and 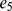 for the above case are presented in Figure 12. Notice the smaller number of concentric rings conforming to the LV in 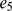. These two edge-gradient pairs are processed to vote into a unique parameter space, only with different weights. Since ARLR is minimized in 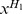, suggesting that the LV has the most influential impact relatively in 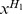, the pair 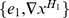 votes with a positive weight, which is selected as 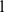 in our study. In the contrast, the pair 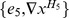 votes with a negative weight (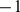) due to the fact that ARLR is maximized in 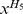. The candidates from 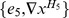 mostly deviate from the LV center. Negative weights for them would largely suppress the incorrect candidates such as the ones in Figure 1. The whole algorithm is presented in Table 2.

**Table 2:** Anisotropic Weighted Circle Hough Transform.

Inputs	
1.	Edge map 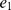 from 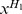 ;
2.	Edge map 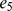 from 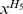 ;
3.	Gradient map 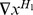 ;
4.	Gradient map 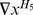 ;
**Algorithm**	
1	Initialize 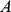 to 0
2	For each 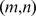 in 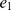
3	If 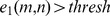
4	For 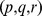 in 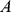 , s.t. 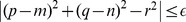
5	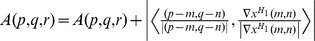
6	End For
7	End If
8	End If
9	For each 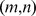 in 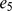
10	If 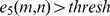
11	For 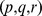 in 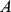 , s.t. 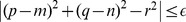
12	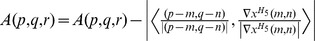
13	End For
14	End If
15	End If
**Outputs** **1.**	**Parameter Space 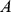 **
Abbreviations:	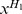 , Harmonic image 1;
	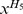 , Harmonic image 5;
	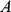 , 3D parameter space;
	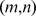 , spatial coordinates;
	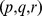 , parameter coordinates;
	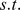 , such that;
	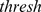 , threshold

**Figure 13:**
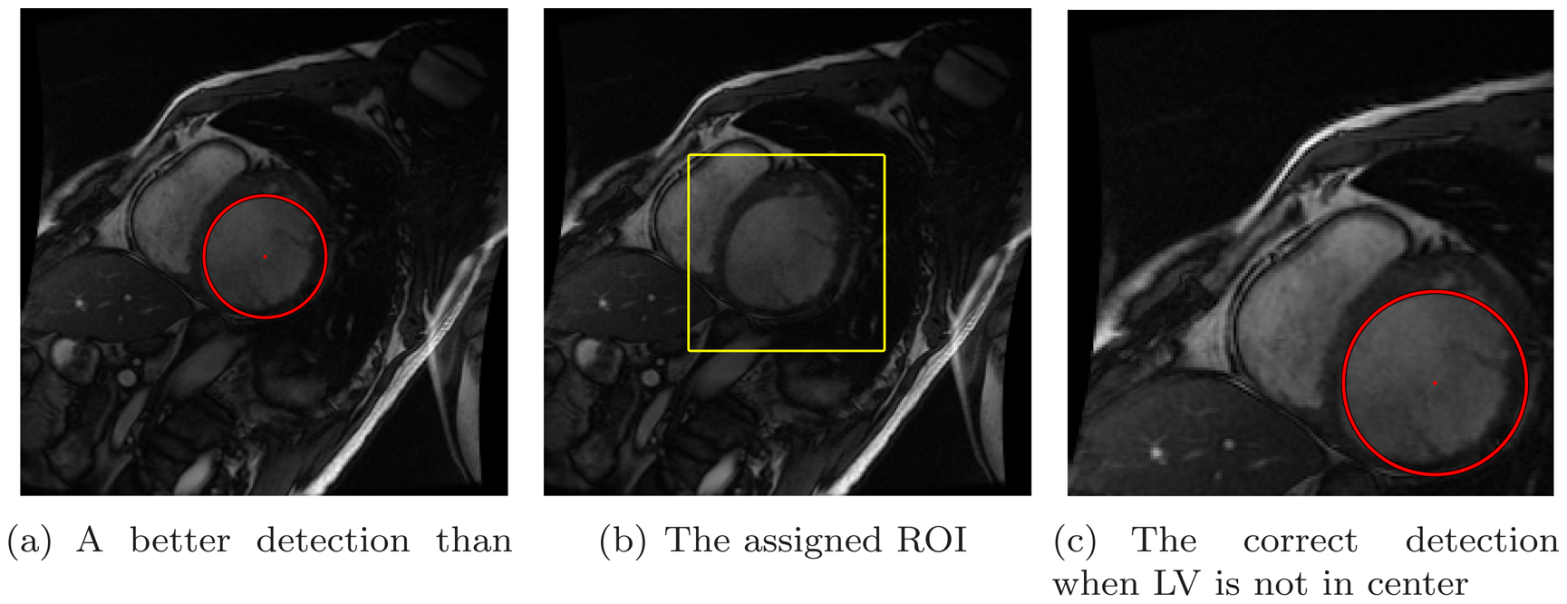
Better detection.

After two voting rounds are completed, the highest accumulator in the 3 D parameter space suggests the LV center as well as the diameter of the LV cavity. We can see a much more precise localization result in Figure 13(a). This could provide a better initialization for edge-based or region-based segmentation approaches. Compared to the approach that counts on the LV-centered assumption in [27], our method is intrinsically rotational and translational invariant. The correct localization result for the cropped cine image, in which LV is far away from the center of FOV, is shown in Figure 13(c).

## Results

**Figure 14:**
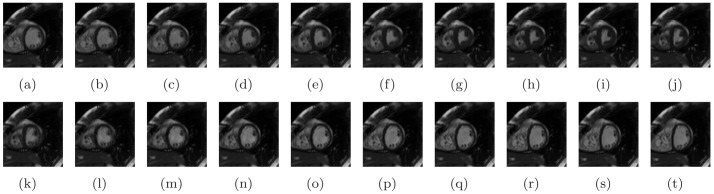
Cropped images for all frames of Volunteer 1.

After the circle detection, a 3 D parameter space 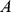 is obtained with 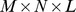 accumulators. The highest accumulator 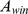 corresponds to the detected circle centered at 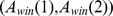 with radius 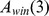. Such a circle is plotted in Figure 13(a) and (c). In practice, the region of interest is identified as the rectangle that bounds this circle with some extension. Such a extended ROI is also plotted in the same case, shown in Figure 13(b) with the expanding ratio set to 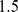 in this study. The cropped images through the whole cardiac cycle are shown in Figure 14. The cine image contains 20 frames, the end-diastole frame of which is shown in the first sub-figure.

The proposed method was tried in 10 volunteer participants, 5 males and 5 females, mean age 40.5, age range from 24 to 59 years. The cine MR images were acquired on a 1.5 T Siemens scanner. The cine images was ECG gated. The slice thickness is 8 mm. The pixel spacing is from 1.25 mm to 1.87 mm, typically 1.71 mm. The TR/TE/flip angle is between 
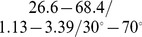
, typically 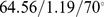. A cine image slice contains typically 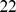 time frames over a cardiac cycle. The algorithm was implemented by Matlab and applied on the basal image sequence of each volunteer case. The computational time for each case averaged 3.94 seconds. Generating Harmonic images took 2.987 seconds, while LV detection took 0.956 seconds.

**Figure 15:**
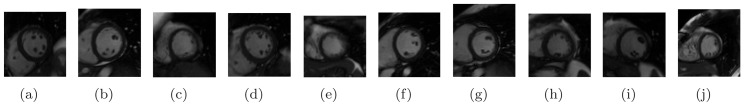
Cropped images for end-diastole frames of ten volunteers.

The cropped ROI of all cases contains the LV, which is visually inspected. The size ratio of the cropped ROI to the whole image ranges from 0.136 to 0.185, average is 0.165, which is a large reduction for the computation load. The statistics of experiments with all participants are shown in Table 3 and the cropped images of the end-diastole frame of all participants are shown in Figure 15.

**Table 3:** Statistics on Experiment Results.

Volunteers	Gender	Age	Original Image Size	Cropped Image Size	Cropping Ratio
1	Male	29	192*162	79*73	0.18541
2	Male	44	192*162	71*69	0.157504
3	Male	46	192*150	66*65	0.148958
4	Female	43	192*150	64*75	0.166667
5	Female	24	192*150	68*71	0.167639
6	Female	59	192*150	62*63	0.135625
7	Male	50	192*150	72*71	0.1775
8	Female	52	192*144	69*67	0.167209
9	Female	33	192*150	69*75	0.179688
10	Male	25	192*150	70*67	0.162847
Average	N/A	40.5	192*152	approx 69*69	0.165

## Discussion

A new spectrum-based computer-aided tool has been proposed and developed for automatic LV localization.We found that the RV presented higher brightness than the LV in all harmonic images. The combination of the first and fifth harmonic images selected for an anisotropic weighted circle Hough detector was found to be the most robust for locating the LV. The development of this technique, which enables the automated location and further segmentation of the LV, will have a significant impact in research and in diagnostic settings, since we envisage that the automated method can be used by radiographers and cardiologists who will perform the diagnosis and assessment of ventricular function in patients with diverse heart diseases, like ischemic dilated cardiomyopathy [28], repaired Tetralogy of Fallot [29], heart failure before and after surgery [30], diastolic heart failure [31], [32].

Only the basal short-axis images were used for analysis in our experiments. We selected the top basal images (i.e., the first slice below the slice containing LV Outflow Tract). The purpose of the automatic localization method proposed in our paper is to provide an initialization for the segmentation. The propagation of a single image segmentation result to other frames or other slices is quite common in approaches for the whole LV segmentation. Hence choosing the best starting slice and time frame is significant for minimizing the propagation error. The top basal images are chosen for the large dimension of both the LV and RV on such slices. In contrast, the LV and RV occupy only small portion of the image and contain a small number of pixels. Any noises would affect the detection precision. A greater viability of harmonic images at apical slices could introduce more difficulties for our algorithm, while containing more pathological implication. Hence, a future study interest of ours is the application of this method on images that have more slice locations.

To clarify, we tested our algorithm on 10 healthy volunteers without any cardiovascular pathology. These 10 subjects constitute the study population used to demonstrate the proof-of-concept of our method. It should be noted that this is the first study to develop the described novel method for automated localization with the aim to simplify segmentation of LV cine images, which might represent a step forward in this field. Since the shape and motion pattern of the left ventricle and right ventricle may vary between patients, a validation study to test the approach in a bigger population with and without pathologies would be a logical next step.

In this study, we conducted the experiments in normal participants. The methodology proposed is also from an image analysis aspect. Furthermore, the relationship between harmonic images and the heart atlas concerning anatomy and functions would be more interesting for clinicians. The analysis of the correlation between harmonic image patterns and pathologies would be a powerful diagnosis aid tool, and this is a future research interest of ours.
